# More is less, less is more, or does it really matter? The curious case of impact of azacitidine administration schedules on outcomes in patients with myelodysplastic syndromes

**DOI:** 10.1186/s12878-018-0095-2

**Published:** 2018-02-01

**Authors:** Rory M. Shallis, Amer M. Zeidan

**Affiliations:** 1 Department of Internal Medicine, School of Medicine, Yale University, New Haven, CT USA; 2 Cancer Outcomes, Public Policy, and Effectiveness Research (COPPER) Center, Yale University, New Haven, CT USA; 3 Section of Hematology, Department of Internal Medicine, Yale University, 333 Cedar Street, PO Box 208028, New Haven, CT 06520-8028 USA

**Keywords:** Myelodysplastic syndrome, MDS, Azacitidine, Decitabine, Hypomethylating agents, HMAs

## Abstract

Myelodysplastic syndromes (MDS) encompass a diverse group of hematologic disorders characterized by ineffective and malignant hematopoiesis, peripheral cytopenias and significantly increased risk of progression to acute myeloid leukemia (AML). The hypomethylating agents (HMA) azacitidine and decitabine induce meaningful clinical responses in a significant subset of patients with MDS. Though never compared directly with decitabine, only azacitidine has improved overall survival (OS) compared to conventional care in a randomized trial in patients with higher-risk MDS. The azacitidine regimen used in this pivotal trial AZA-001 included administration at 75 mg/m^2^/day for 7 consecutive days in 28-day cycles (7–0 regimen). Given the logistical difficulties of weekend administration in the 7–0 regimen, as well as in efforts to improve response rates, alternative dosing schedules have been used. In a typical 28-day cycle, administration schedules of 3, 5, 10, and (with the oral version of azacitidine) 14 and 21 days have been used in clinical trials. Most trials that evaluated alternative administration schedules of azacitidine did so in lower-risk MDS and did not directly compare to the 7–0 schedule. Given the lack of randomized prospective studies comparing the 7–0 schedule to the other regimens of azacitidine in MDS, Shapiro et al. conducted a systematic review in an attempt to answer this question. Here we place the findings of this important work in clinical context and review the current knowledge and unresolved issues regarding the impact of administration schedules of azacitidine on outcomes of patients with both lower-risk and higher-risk MDS.

## Background

Myelodysplastic syndromes (MDS) comprise a heterogenous subset of the myeloid malignancies and are characterized by disordered and malfunctional hematopoiesis, subsequently leading to clinically significant cytopenias and a substantial risk of progression to acute myeloid leukemia (AML) [1]. Treatment with the hypomethylating agents (HMAs) azacitidine (5-azacytidine) and decitabine (5-aza-2′-deoxycytidine) leads to objective clinical responses in nearly half of patients with MDS, and delays progression to AML [2–6]. The two HMAs have never been prospectively compared head-to-head with regards to overall response rate (ORR; the sum of complete response [CR], partial response [PR], and hematological improvement [HI] according to the International Working Group (IWG) 2006 criteria) or their effect on survival [7].

## Main text

The early phase cancer and leukemia group B [CALGB] trials demonstrated favorable responses to azacitidine in about half the patients [Table 1]; many of them had excess blasts [2, 3]. The CALGB9221 trial was the first randomized phase III trial of azacitidine in MDS, and showed that azacitidine had an ORR of 60% and delayed progression to AML compared to best supportive care [BSC] [5]. Although there was no impact on overall survival, potentially because the trial allowed early cross-over to azacitidine, the drug was approved for treatment of MDS in the United States in 2004. The landmark AZA-001, published in 2009, was the first and only randomized phase III trial to demonstrate an OS advantage with a drug (azacitidine, *N* = 179) compared to conventional care regimens (CRR, N = 179, which included pre-selected BSC [59%], low-dose cytarabine [27%], or intensive chemotherapy [14%]) in patients with international prognostic scoring system (IPSS) intermediate-2 or high risk MDS (24.5 months vs. 15.0 months; hazard ration = 0.58, *p* = 0.0001) [4]. The survival advantage was confirmed in each subset of response, including those whose best response was HI [8]. Based on the results of this seminal study, the European Medicines Agency approved azacitidine for use in higher-risk MDS (i.e. IPSS intermediate-2 and high risk). Both phase 3 studies used the standard schedule of 7 consecutive days of azacitidine at 75/m^2^/day in 28-day cycles (the so called 7–0 regimen). While decitabine is also approved in USA for use in MDS based on improvement in response rates and delayed progression to AML, neither of the two large randomized trials that compared decitabine to BSC in MDS showed a significant difference in OS [6, 9].

**Table 1:** Selected prospective studies using diffirent azacitidine administration schedules in myelodysplastic syndrome

Regimen(s) studied	Comparator	Type	MDS risk	N	Results	Reference
75 mg/m^2^ × 7 days continuously	None	Phase II	98% HR, 0% LR	43	ORR 49%, CR 15%, median OS 13.3 months	Silverman, et al. [2] (CALGB 8421)
7–0	None	Phase II	50% HR, 16% LR	67	ORR 52%, CR 17%	Silverman, et al. [3] (CALGB 8921)
7–0	BSC	Phase III	46% HR, 54% LR	191	ORR 60%; OS: AZA 20 months vs. BSC 14 months, *p* = 0.10 (53% of BSC group crossed over to receive AZA)	Silverman, et al. [5]
7–0	CCR	Phase III	100% HR	358	ORR 49%; CR 17%; OS: AZA 24.5 months AZA vs. CCR 15 months, HR 0.58 (95%CI 0.43–0.77, *p* = 0.0001)	Fenaux, et al. [4] (AZA-001)
5–2-2 vs. 5–2-5* vs. 5–0	N/A	Phase III	48% LR, 52% HR	103	HI rates of 44%, 45%, and 56%, respectively; TI rates of 50%, 55%, and 64%, respectively; CR and OS not assessed	Lyons, et al. [10]
7–0	N/A	Phase II	59% HR, 41% LR	22	ORR 27%, CR 27%, OS not assessed	Martin, et al. [37]
50 mg/m^2^/d × 10 days	AZA + entinostat 4 mg/m^2^/d day 3 + day 10	Phase II	45% HR, 18% LR	97	ORR 46% on AZA only arm, including unilineage HI; CR 12%; median OS 18 months	Prebet, et al. [27] (E1905)
CC-486 (oral AZA) 120-600 mg daily	None	Phase I	48% HR, 48% LR	29	ORR 73% and CR 40% for treatment-naïve, ORR 35% and CR 0% for previously-treated (including CMML)	Garcia-Manero, et al. [29]
5–2-2	N/A	Prospective, observational	100% HR	38	ORR 47%, CR 18%, median OS 16.4 months	Breccia, et al. [33]
5–2-2	N/A	Prospective, observational	100% HR	60	ORR 63%, CR 25%, median OS 17 months	Breccia et al. [34]
5–0	N/A	Phase II	100% LR	32	ORR 47% on intention-to-treat, CR 19%, median OS 28.5 months	Fil, et al. [35]
5–0	N/A	Phase II	100% LR	30	ORR 20% (included possible ESA use), CR not reported, median OS not reached	Tobbiason, et al. [38]
CC-486 (oral AZA) 300 mg daily × 14 days or 21 days	N/A	Phase I/II	100% LR (27% by IPSS-R)	55	14-day arm: ORR 36%, CR 14%; 21-day arm: OR 41%, CR 0%; OS not assessed	Garcia-Manero, et al. [30]
75 mg/m^2^ × 3 days	Decitabine 20 mg/m^2^ × 3 days	Phase II	100% LR (20% HR by IPSS-R)	40	ORR 49%, CR 36%, median OS not reached	Jabbour, et al. [32]

*Abbreviations*: *AZA* Azacitidine, *BSC* Best supportive care, *CCR* Conventional care regimen, *CI* Confidence interval, *CMML* Chronic myelomonocytic leukemia, *CR* Complete response, *HI* Hematological improvement, *HR* Higher-risk, *LR* Lower-risk, *ORR* Overall response rate, *OS* Overall survival, *RCT* Randomized controlled trial, *SD* Stable disease, *TI* Transfusion-independence

*5–2-5: azacitidine 50 mg/m^2^ daily subcutaneously for 5 days, then 2 days without therapy, then 50 mg/m^2^ daily subcutaneously for 5 days

The approved regimen of azacitidine (7–0) requires weekend administration and poses logistical challenges to both infusion centers and patients. The obligation to arrange weekend administration is difficult for community practices and even well-resourced institutions which can be burdened with increased cost, limitations in staffing, and patient preferences for more convenient options. Infusion site reactions and bruising are also fairly-common. Given these complications, alternative administration schedules that avoid weekend administration have been developed and evaluated. In the only randomized phase 2 trial to compare different azacitidine administration schedules in MDS, 151 patients were randomized in community-based centers to one of three weekend-less subcutaneous regimens: azacitidine 75 mg/m^2^ daily for 5 days, followed by 2 days without treatment, then 75 mg/m2 daily for 2 days (or 5–2-2); azacitidine 75 mg/m^2^ daily for 5 days (5–0); or azacitidine 50 mg/m^2^ daily for 5 days, followed by 2 days without treatment, then 50 mg/m^2^ daily for 5 days (or 5–2-5) [10]. There were no significant differences in the HI rates (44%, 56%, and 45%) or in rates of red blood cell (RBC) transfusion-independence (50%, 64%, and 55%) between 5 and 2-2, 5–0, and 5–2-5 groups, respectively. Importantly, most studied patients (63%) in the trial had lower-risk MDS as defined by the French-American-British (FAB) classification, namely refractory anemia, refractory anemia with ringed sideroblasts, and chronic myelomonocytic leukemia with <5% bone marrow blasts [10, 11]. Since a minority of patients in this study had higher-risk MDS, there was no arm which used the standard 7–0 regimen, and there were no survival data in this study, these results cannot reliably be extrapolated to guide therapy in higher-risk MDS patients in whom the 7–0 regimen remains the standard of care [10].

In their recent study, Shapiro et al. conducted a systematic review of the available data comparing the different azacitidine administration schedules used for treatment of MDS, explicitly the 5–0, 5–2-2, and 7–0 regimens [12]. Attempts at conducting a meta-analysis were unsuccessful given the paucity of randomized controlled studies (4 of 130 included studies, or 3%) evaluating differences in administration schedules and the significant heterogeneity of the observational studies, but an ORR for each regimen was obtained via pooled analyses of the included studies. Two observational retrospective studies which compared the 5–0 with the 7–0 schedule were also included, but in only one of two studies the study of patients had higher risk MDS and neither study showed a significant difference in OS [13, 14]. The pooled proportion analyses of ORR were 44.4% with 95% CI (42.4%–45.1%) for the 7–0 schedule, 41.2% with 95% CI (39.2%–41.9%) for the 5–0 schedule, and 45.8% with 95% CI (42.6%–46.4%) for the 5–2-2 schedule. Shapiro et al. suggest that schedules administering seven days of azacitidine treatment with or without a weekend break might have higher ORR than the other five-day schedules, but the fact that pooled results of alternative regimens cannot be directly compared limits this conclusion especially in patients with higher risk MDS. Shapiro et al. duly caution that their analysis was limited by several significant biases, the heterogeneity in design of the studies included (most of which were retrospective or uncontrolled) as well as their reporting of metrics (namely ORR and OS but not CR), which precluded direct comparisons between schedules [12].

Most studies that have used the 5–0 azacitidine regimen, including the only randomized prospective study comparing differing schedules by Lyons et al., were comprised of a clear majority of lower-risk MDS patients [Table 1] [10]. It is also worth noting that the observed effect on survival as demonstrated in the influential AZA-001 trial has not been replicated in real-world or population studies of higher-risk MDS patients. For example, a study of one of the largest reported cohort of patients with HMA-treated higher-risk MDS in the USA demonstrated that the median OS for patients treated with azacitidine was 16.4 months (95% CI, 15.0–17.9 months) [15]. Another study using the Surveillance, Epidemiology, and End Results (SEER)-Medicare linked database showed that median OS with azacitidine among older patients with refractory anemia with excess blasts was 11 months [16]. One theory that has been proposed to explain the substantial difference in survival between real-life analyses and AZA-001 for patients with higher-risk MDS treated with azacitidine is the significant underuse of the 7–0 regimen in the community setting in favor of alternative administration schedules in the USA [17]. Indeed, one registry in the USA suggested that the 7–0 regimen is only used 15% of the time [18]. However, registry and population studies from Europe, in which patients often receive the approved 7–0 regimen, also reported significantly worse survivals than AZA-001 [14, 19, 20]. Therefore, it is likely that other factors aside from the administration schedule account for the substantially worse performance of azacitidine in the real-life setting in patients with higher-risk MDS [4, 19, 21–23].

On a mechanistic level, there are no well-established minimal effective doses for HMAs, including azacitidine [24]. The precise mechanism of action of azacitidine in MDS remains poorly defined despite extensive research and clinical use of the drug for almost 15 years now [25]. While, as its class name implies, epigenetic modification and hypomethylation are central to the prevailing theory regarding their mechanism of action, immune-mediated and other effects might be in play [26]. Incorporation of azacitidine in RNA and DNA appear central to its activity, and with its short half-life combined with the need for the cells to be in the S-phase of the cell cycle for the drug to be incorporated into the DNA, it has been proposed that prolonged exposures to azacitidine via longer administrated schedules might be better pharmacodynamically in increasing the chance of targeting slow-cycling MDS cells [27]. Lower-dose, but prolonged schedules of azacitidine at 50 mg/m^2^ via the 5–2-5 schedule has been previously shown to effectively reverse promoter methylation [28]. Clinical metrics were also validated by the US Leukemia Intergroup Randomized Phase Study E1905 in which the 5–2-5 schedule resulted in a targeted 30% trilineage hematologic response rate, approximately double that seen in the AZA-001 study [27]. However, the survival associated with this regimen appeared worse than that of the 7–0 regimen in the AZA-001 trial, keeping in mind all the problems that arise in cross-trial comparisons. A phase I study of the oral formulation of azacitidine (CC-486) administered via the 7–0 schedule to a majority of lower-risk MDS patient has demonstrated an ORR of 73% and favorable side effect profile [29]. Fourteen and 21-day administration schedules of CC-486 have also demonstrated substantial ORR [30]. Perhaps avoiding the infusion requirement is the more convenient approach to deliver longer exposure to azacitidine, of course presuming a comparable or superior efficacy. A phase III multi-center randomized double-blind study is currently recruiting lower-risk MDS patients to be treated with the oral formulation using a prolonged administration schedule [31]. To further cloud the proposition that extended administration and thus extended exposure translates to better outcomes, a recent study has suggested that a shorter, three-day course of azacitidine for lower-risk MDS results in a significant ORR and CR rates of 49% and 36%, respectively [32]. This grossly parallels the ORR and CR rates noted in prospective studies specifically evaluating 5-day regimens and the approved 7-day regimens, however there has been no direct comparison to the 7–0 regimen and patients with higher-risk MDS were not treated in this trial [2–5, 33–35].

## Conclusions

In summary, inconsistent data with regards to the optimal use and schedules of azacitidine in both higher-risk and lower-risk MDS is leaving a lot of uncertainty regarding the best ways to administer this drug. When put in the context of clinical considerations and logistical difficulties, extended azacitidine administration regimens (e.g. 7–0) are often cumbersome. However, given the lack of convincing data otherwise, the approved 7–0 azacitidine schedule remains the only regimen associated with a survival advantage and is the recommended treatment for higher-risk MDS [1, 36]. While the 5–2-2 regimen is considered by many experts to be equivalent to the 7–0 regimen based on indirect comparison data [33, 34], there are no randomized prospective data comparing to 7–0 regimen to support that notion. On the other hand, in lower risk MDS where the focus is on quality of life improvement and where no drug or regimen has prolonged survival, alternative (shorter or potentially longer) azacitidine regimens are generally appropriate. Ultimately, randomized clinical trials directly comparing azacitidine administration schedules in both lower risk and higher risk MDS patients are needed to guide the best practice in the community.

## Abbreviations

AML: Acute myeloid leukemia
BSC: Best supportive care
CALGB: Cancer and leukemia group B
CI: Confidence interval
CR: Complete response
FAB: French-American-British
HI: Hematological improvement
HMA: Hypomethylating agent(s)
IPSS: International prognostic scoring system
IWG: International Working Group
MDS: Myelodysplastic syndrome(s)
ORR: Overall response rate
OS: Overall survival
PR: Partial response improvement
RBC: Red blood cell
SEER: Surveillance, epidemiology, and end results
USA: United States of America
